# Iron deposition is associated with differential macrophage infiltration and therapeutic response to iron chelation in prostate cancer

**DOI:** 10.1038/s41598-017-11899-2

**Published:** 2017-09-14

**Authors:** Avigdor Leftin, Huiyong Zhao, Mesru Turkekul, Elisa de Stanchina, Katia Manova, Jason A. Koutcher

**Affiliations:** 1 Department of Medical Physics, Memorial Sloan Kettering Cancer Center, New York, NY USA; 2 Antitumor Assessment Core Facility, Memorial Sloan Kettering Cancer Center, New York, NY USA; 3 Molecular Cytology Core Facility, Memorial Sloan Kettering Cancer Center, New York, NY USA; 4 Department of Medicine, Memorial Sloan Kettering Cancer Center, New York, NY USA; 5 Molecular Pharmacology Program, Memorial Sloan Kettering Cancer Center, New York, NY USA

## Abstract

Immune cells such as macrophages are drivers and biomarkers of most cancers. Scoring macrophage infiltration in tumor tissue provides a prognostic assessment that is correlated with disease outcome and therapeutic response, but generally requires invasive biopsy. Routine detection of hemosiderin iron aggregates in macrophages in other settings histologically and *in vivo* by MRI suggests that similar assessments in cancer can bridge a gap in our ability to assess tumor macrophage infiltration. Quantitative histological and *in vivo* MRI assessments of non-heme cellular iron revealed that preclinical prostate tumor models could be differentiated according to hemosiderin iron accumulation—both in tumors and systemically. Monitoring cellular iron levels during “off-label” administration of the FDA-approved iron chelator deferiprone evidenced significant reductions in tumor size without extensive perturbation to these iron deposits. Spatial profiling of the iron-laden infiltrates further demonstrated that higher numbers of infiltrating macrophage iron deposits was associated with lower anti-tumor chelation therapy response. Imaging macrophages according to their innate iron status provides a new phenotypic window into the immune tumor landscape and reveals a prognostic biomarker associated with macrophage infiltration and therapeutic outcome.

## Introduction

The extent of immune cell infiltration in tumors is correlated with predicted therapeutic response and survival probability in many cancers, including prostate cancer (PCa)^1^. Macrophage infiltration specifically is recognized as a significant negative contributing factor to PCa in patients and animal models^2–6^. Efforts to identify factors associated with the infiltrative pro-tumor behavior of macrophages have revealed numerous cytokine-signaling networks that regulate their function in the tumor microenvironment^7–9^. Many of these fundamental insights into macrophage immune response have led to new immune therapies, which have shown promise in preclinical PCa models^10, 11^. However, most of these therapeutic targets do not have corresponding endogenous, non-invasive *in vivo* imaging biomarkers requiring surrogate measures of treatment response often restricted to bulk imaging assessment of tumor burden, and measures of immune cells by invasive biopsy.

In addition to their function in the innate immune response, macrophages also play key roles in recycling iron^12–14^. This is facilitated by the sequestration of iron(III) in ferritin protein aggregates known as hemosiderin in so-called hemosiderin laden macrophages (HLMs) that prevent depletion of limited body iron stores, and contribute to maintaining low equilibrium levels of cytotoxic chelatable free iron^12, 15, 16^. The ability of macrophages to store and redistribute iron positions them uniquely to regulate the bioavailability of iron to tumor cells, by serving as local metabolic deposits fueling tumor growth^8, 9^. To target this driver of cancer, “off-label” administration of small molecule iron(III) chelators used clinically to control inherited or transfusion-dependent cellular iron overload have been tested^17–21^. Iron(III) chelators such as the FDA-approved drug deferiprone (L1) have been shown to inhibit cancer cell growth through a variety of mechanisms such as involving inhibition of iron-dependent translational and enzymatic processes^17, 18, 22–30^.

Hemosiderin has long been known to be a specific *in vivo* MRI biomarker of macrophages^31–33^. Aggregated hemosiderin deposits in HLMs contain solid crystalline superparamagnetic iron (>10 wt. %^34^) that contributes to high T_2_^*^ MRI contrast relative to other soluble paramagnetic bio-iron sources such as plasma transferrin, ferritin, and (deoxy) hemoglobin^35–39^. T_2_^*^ MRI techniques have been implemented clinically as iron MRI methods used as a replacement for tissue iron biopsy in iron overload disorders of the liver, heart, and brain^40–43^. These methods are generally employed to measure levels of tissue iron prior to chelation therapy as a predictor of chelation efficacy, and monitor these cellular iron levels during treatment to determine therapeutic response and guard against over-chelation^44–47^. This suggests that quantitative iron MRI can also be used to score HLM infiltration in the tumors, as well as provide an intrinsic *in vivo* cellular biomarker for targeted chelation cancer therapy.

Cellular iron levels in normal tissues also vary with genetic background of the mouse strain^48^. However, quantitative *in vivo* and histological imaging studies have not addressed associations between macrophages and iron accumulation as a function of background, although macrophage iron content and accumulation in pathological tissues presents them as prime therapeutic imaging targets that have been associated with clinical chelation efficacy in other settings^47, 49^. Importantly, these studies also have not been carried out in cancer, leaving a gap in our understanding of factors associated with anti-tumor iron chelation response. Thus, we chose the Myc-CaP and TRAMP-C2 transgenic prostate cancer cell lines as two common orthotopically implantable *in vivo* models differing generally in iron background due to their syngeneic FVB/N and C57BL/6 syngeneic mouse hosts^50^ in order to correlate the spatial distributions of iron deposits with systemic and anti-tumor response to chelation therapy in prostate cancer.

## Results

**Figure 1:**
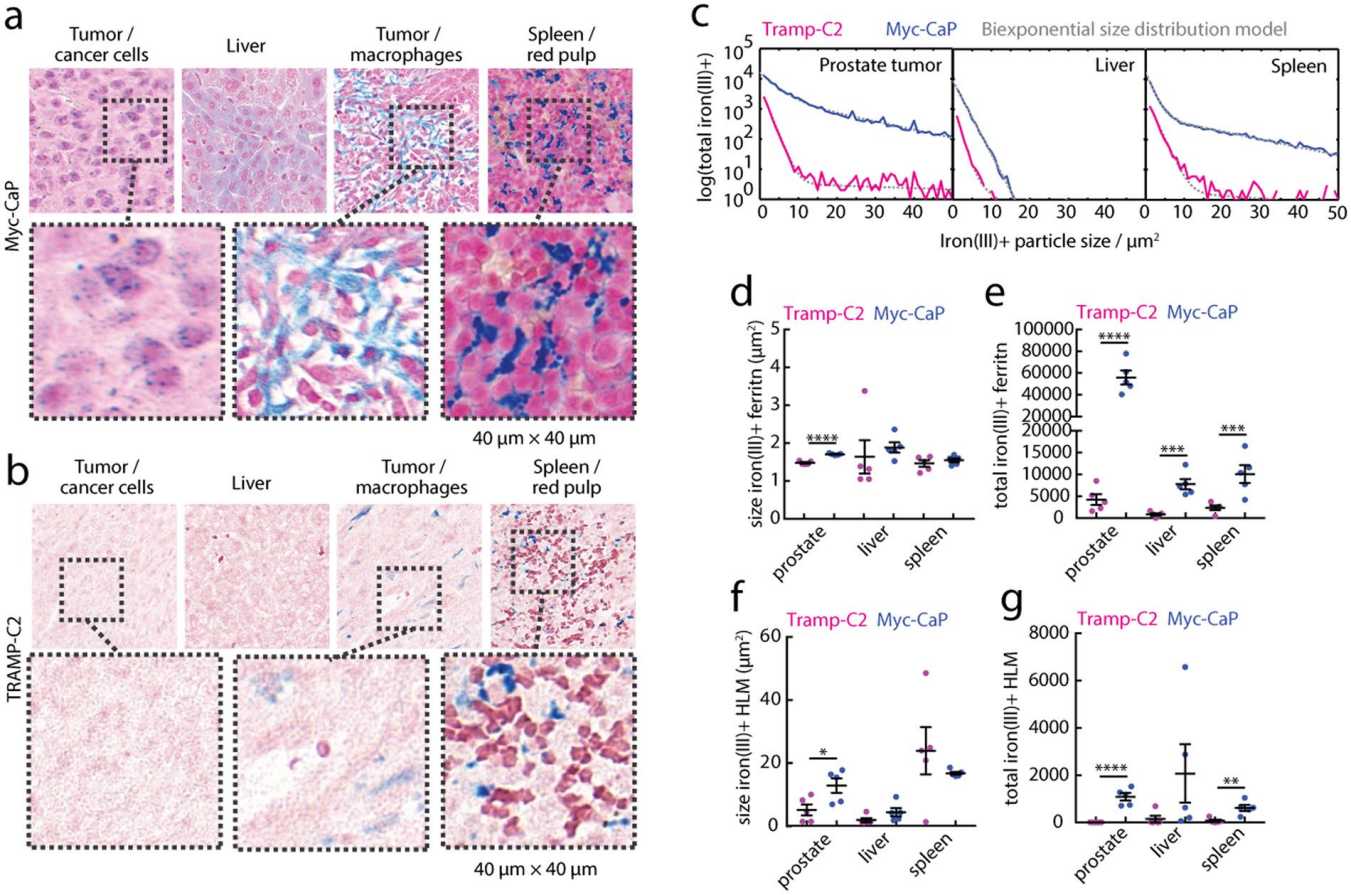
Non-heme cellular iron(III) in systemic and prostate tumor microenvironments. Prussian blue iron(III) histology of (**a**) Myc-CaP and (**b**) TRAMP-C2 prostate cancer model tumor, liver, and spleen. Expansion scale 40 μm × 40 μm. Two general classes of particles were observed; small ferritin granule aggregates (fer), and large hemosiderin laden macrophages (HLMs). (**c**) The total iron(III)^+^ positive features of whole tissue cross-sections counted in the cross-section plotted as a function of iron(III)^+^ particle size (solid lines), and biexponential size distribution model (dashed lines). Particle areas and their population size for (**d**,**e**) fer, and (**f**,**g**) HLMs from the model fits, respectively (mean ± s.e.m; n = 5 per tissue, per model; *p < 0.05, **p < 0.01, ***p < 0.001, ****p < 0.0001, two-tailed unpaired t-test).

To first assess cellular associations of iron(III) deposits systemically and in the tumor microenvironment, we performed Prussian Blue histochemistry and quantified the total non-heme cellular iron(III) content in tumors, livers, and spleens of tumor-bearing Myc-CaP (Fig. 1a) and TRAMP-C2 (Fig. 1b) mice. First inspection of the histological tissue sections revealed small ferritin granules (fer) in the proximity of Myc-CaP prostate cancer cells and liver hepatocytes and Kuppfer cells, but these particles were rare in the equivalent TRAMP-C2 tissues. Also present in the Myc-CaP tissues were cell-sized iron(III)^+^ particles, characterized as HLMs, found clustered in the tumor, occasionally in Kuppfer cells in the liver, and prominently in the red-pulp in the spleen. Similar to the small fer granules, the HLM deposits were found abundantly in Myc-CaP tissues, but were hardly detectable in tissues of the TRAMP-C2 mice.

To fully quantify these observations, we used an automated image feature detection algorithm to count the total number of iron(III)^+^ particles and measure their sizes in whole tissue cross sections of prostate tumors, livers, and spleens of the models from the iron(III)^+^ particle size distributions (Fig. 1c). The fer granules were larger in Myc-CaP prostate tumors (p < 0.0001, Fig. 1d), and similarly HLMs were larger in prostate tumors of the Myc-CaP model (p < 0.05, Fig. 1f), while both the size of fer granules and HLMs were similar in livers and spleens of the Myc-CaP and TRAMP-C2 animals (p > 0.05). The total number of the fer iron deposits (Fig. 1e) was also markedly higher in prostate tumors (p < 0.0001), livers (p < 0.001), and spleens (p < 0.001) of the Myc-CaP model compared to the TRAMP-C2, and the amount of HLMs (Fig. 1g) was also significantly more abundant in all the Myc-CaP prostate tumors (p < 0.0001), livers (p < 0.05), and spleens (p < 0.01) in these comparisons. This demonstrates that the two PCa models exhibit strikingly different levels of macrophage iron both systemically in iron-metabolizing organs such as the liver and spleen, as well as in prostate tumors.

**Figure 2:**
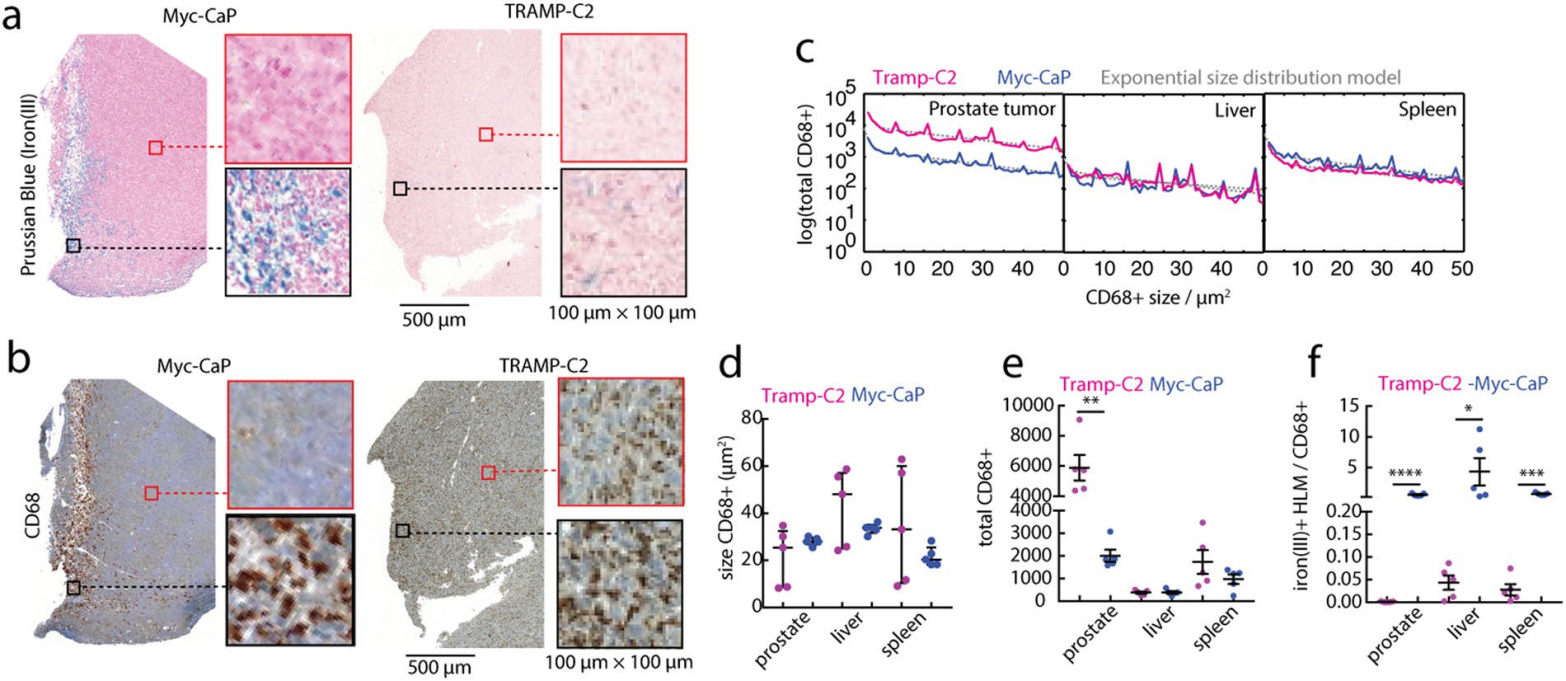
Spatial association of iron(III) and CD68 macrophages in PCa models. (**a**) Prussian blue iron(III) histology and (**b**) CD68 macrophage histology of orthotopic Myc-CaP and TRAMP-C2 prostate tumors. Scale bar 500 μm, and expansion box scale 100 μm × 100 μm. (**c**) Total CD68^+^ macrophages counted in whole tissue cross-sections, plotted (solid lines) as a function of their size in prostate tumors, livers, and spleens in Myc-CaP and TRAMP-C2 models, and exponential size distribution model (dashed lines). (**d**) Size of CD68^+^ macrophages analyzed from the distribution fitting, (**e**) their total frequency in the tissue, and (**f**) the calculated ratio of iron(III)^+^ HLMs to CD68^+^ macrophages (mean ± s.e.m; n = 5 per tissue, per model; *p < 0.05, **p < 0.01, ***p < 0.001, ****p < 0.0001, two-tailed unpaired t-test).

We further characterized the spatial distribution of the HLMs in the tumor and compared this non-standard tumor macrophage marker (iron(III)) with a common macrophage immunohistochemical marker, CD68. We prepared serial slices of whole tumor cross-sections of the Myc-CaP and TRAMP-C2 tissues and stained them with either Prussian blue iron(III) to detect HLMs (Fig. 2a), or with CD68 marker (Fig. 2b). Inspection of the images readily revealed infiltrating clusters of iron(III)^+^ HLMs corresponding to CD68^+^ macrophages in the Myc-CaP tumor sections. By contrast, in the TRAMP-C2 tumors the CD68^+^ macrophages were found abundantly both at the tumor margins and within the tumors, although the iron(III)^+^ HLMs were scarce and when found were localized to outer margins of the tissue.

To quantify these differences in tissue distributions of CD68^+^ macrophages, we again applied the histological image and size distribution analysis algorithms to the CD68-stained whole prostate tumor, liver, and spleen cross-sections. The CD68^+^ macrophage quantity vs. size distribution was quantified using exponential modeling (Fig. 2c), and showed the sizes of the CD68^+^ macrophages were similar in both model’s tissues (Fig. 2d), and were more numerous in TRAMP-C2 prostate tumors (p < 0.01), while their frequency in livers and spleens was equivalent (p > 0.05) between the two models (Fig. 2e). To evaluate the difference in frequency of iron(III)^+^ and CD68^+^ macrophages, we calculated the ratio of the two biomarkers (Fig. 2f). In all tissues analyzed, the iron(III)^+^ and CD68^+^ were observed at approximately the same ratio in the Myc-CaP mice, while the TRAMP-C2 tissues invariably showed a near zero ratio, indicating significant bias towards iron-free macrophages in the tumors (p < 0.0001), livers (p < 0.05) and spleens (p < 0.001).

**Figure 3:**
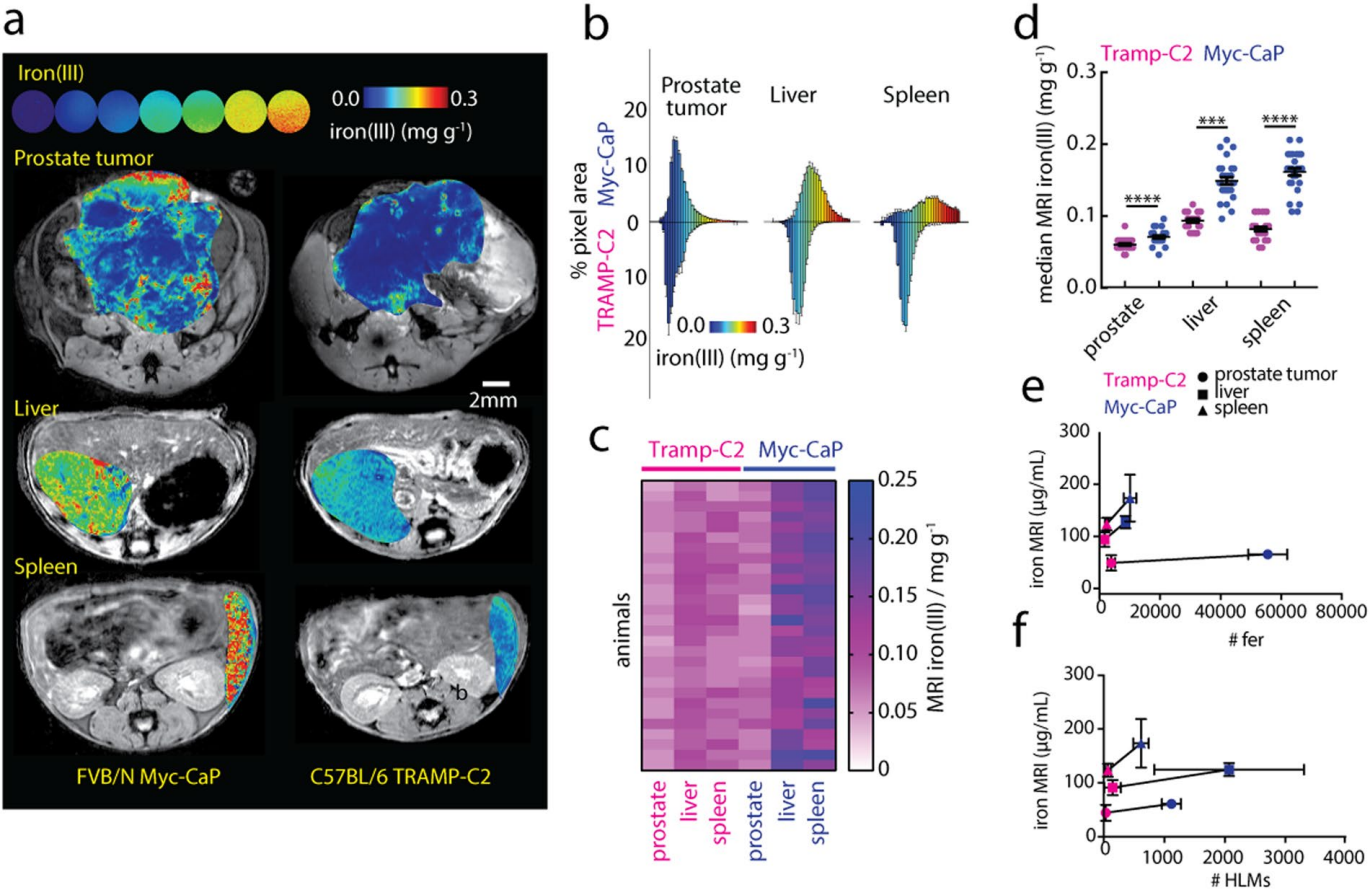
*In vivo* MRI of cellular iron(III) of PCa models. (**a**) Iron MRI maps of aqueous iron solutions (upper) (linear colormap, 0.00–0.30 mg iron(III) g^-1^), and *in vivo* in prostate tumors, livers, and spleens of (left) Myc-CaP, and (right) TRAMP-C2 (right) models. Scale bar 2 mm. (**b**) Parametric pixel distributions of iron(III) (% pixel area vs. iron concentration) in Myc-CaP and TRAMP-C2 model tissues. Median MRI iron(III) levels determined from the pixel distributions and evaluated (**c**) qualitatively by heatmap, and (**d**) quantitatively by statistical comparison (mean ± s.e.m; n = 28 mice per tissue, per model; ***p < 0.001, ****p < 0.0001, two-tailed unpaired t-test). *In vivo* median iron(III) MRI values (n = 14 mice/tissue, mean + /−s.e.m.) for prostate tumors (⚫, ~1 cm^3^), livers (▪) and spleens (▴) as a function of (**e**) average number of ferritin granules and (**f**) HLMs in and TRAMP-C2 models (n = 5 mice per tissue, mean + /−s.e.m). Linear regression (solid-line) between iron MRI measurements in each respective tissue with the number of cellular iron particles in the tissues. (Tumor HLM: y = 0.015x + 44.11, tumor fer: y = 0.00031x + 42.85; liver HLM: 0.017x + 87.42, liver fer: y = 0.0048x + 85.92; spleen HLM y = 0.091x + 117.3, spleen fer: y = 0.006x + 108.4).

The measurement of endogenous cellular iron(III) by T_2_^*^ MRI relaxometry is a standard clinical test performed to assess tissue levels of cellular iron, predominantly in the liver where it is a quantitative biomarker of hereditary or transfusion-dependent iron-overload^46, 51^. We explored the use of this MRI technique to measure cellular iron throughout the livers and spleens (both not bearing tumors), and prostate tumors of the Myc-CaP and TRAMP-C2 mouse models (Fig. 3a). The iron(III) maps revealed striking differences in iron(III) levels between the two models, and between the different tissues. To enable quantitative comparison of these observations we reconstructed the pixel distributions of iron(III) contrast levels from whole tissue cross-sectional regions of interest (Fig. 3b), and the distributions were analyzed to calculate the median iron(III) contrast levels in the prostate tumors, livers, and spleens of all the animals (Fig. 3c). As in the histological assessments of iron in the tissues, median MRI iron(III) levels of FVB/N hosts of the Myc-CaP model were significantly higher than in the C57BL/6 of the TRAMP-C2 models in prostate tumors (p < 0.0001), as well as in livers (p < 0.001) and spleens (p < 0.0001) of all animals (Fig. 3d). As an estimate for the relative contribution of the fer granules and HLMs to the MRI contrast in the tumors, livers, and spleens, we performed a simple linear regression using “low-iron” TRAMP-C2 and “high-iron” Myc-CaP iron(III) MRI medians, and average histological cellular iron(III) counts in those tissues (Fig. 3e,f). A positive trend was observed between the MRI iron levels and both fer granule and HLM counts, but slope of the line, that is the ratio of the difference between the high and low iron MRI levels to the difference in fer or HLM counts over were an order of magnitude larger for HLMs. This demonstrates the greater sensitivity of the MRI method for the larger HLM deposits compared with an equivalent number of small fer granules, and indicates that iron MRI can quantify differences in macrophage accumulation in systemic tissues and in the PCa tumors according to endogenous hemosiderin load.

**Figure 4:**
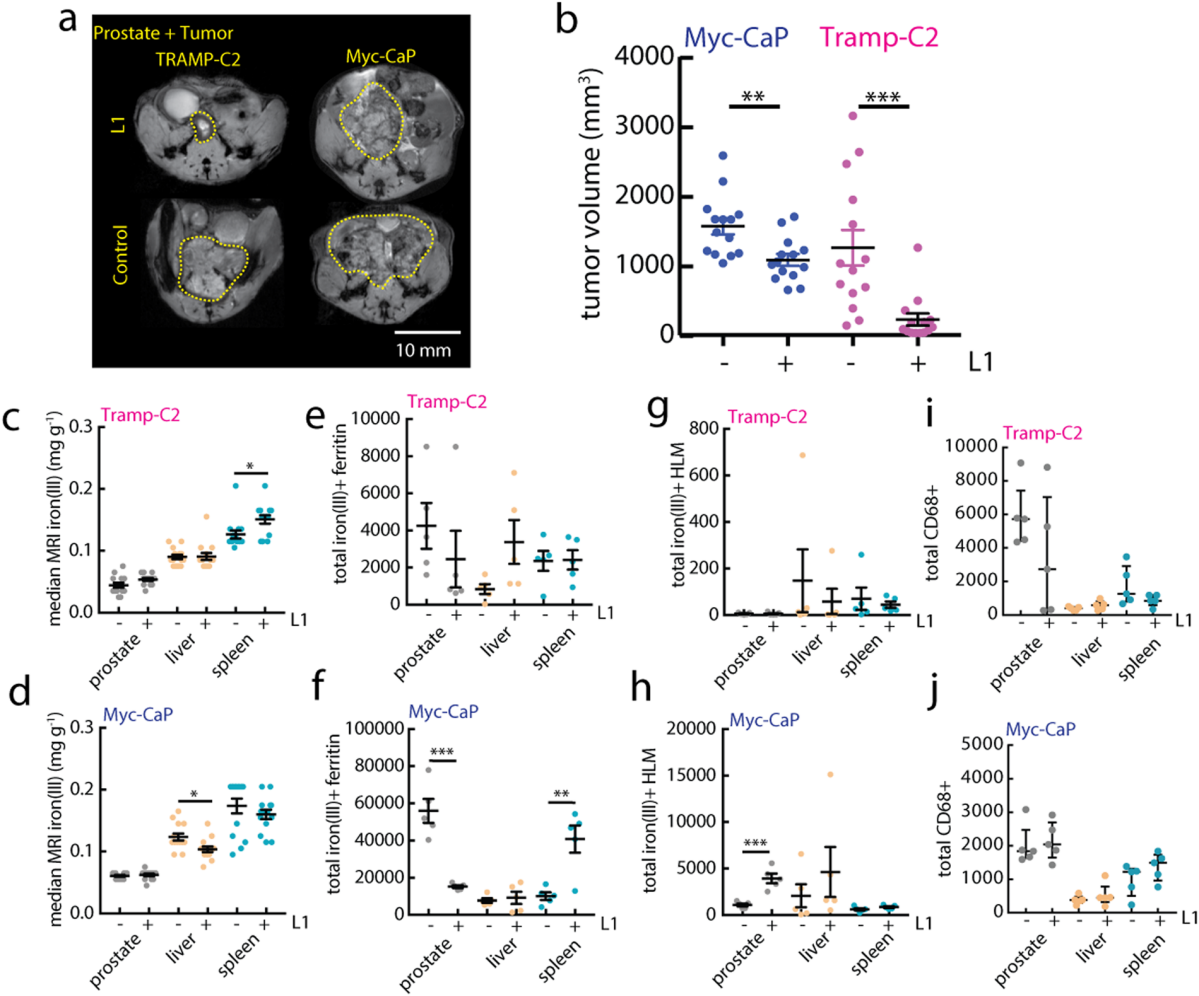
Anti-tumor and non-heme cellular iron response to chelation therapy. (**a**) *In vivo* T_2_^*^-weighted MRI measurements of orthotopic prostate tumor for control and L1 treated (150 mg/kg) Myc-CaP and TRAMP-C2 models at study endpoint. Scale bar 10mm. (**b**) Endpoint tumor volumes for the two models and treatment groups calculated from the MRI images (mean ± s.e.m; n = 14 mice per model; **p < 0.01, ***p < 0.001, two-tailed unpaired t-test). Control (−) and L1-treated ( + ) median iron(III) (**c**,**d**) MRI iron levels measured at study endpoint (n = 14 mice per model; *p < 0.05 two-tailed unpaired t-test), (**e**,**f**) total ferritin granules, (**g**,**h**) total iron(III)^+^ HLMs, and (**i**,**j**) total CD68^+^ macrophage frequency for TRAMP-C2 and Myc-CaP models, respectively (mean ± s.e.m; n = 5 mice per model; *p < 0.05, **p < 0.01, ***p < 0.001, two-tailed unpaired t-test).

We then evaluated the feasibility of using these quantitative iron MRI and histological approaches in a preclinical trial with the iron(III) chelator deferiprone (L1). Chelators such as L1 are prescribed in cases of iron overload, and these chelators administered over months-years exhibit therapeutic efficacy profiles mediated by initial iron(III) levels^47, 49^. We therefore hypothesized that the Myc-CaP model would respond less favorably to L1 therapy, because they have higher numbers of HLMs systemically and in the target tumors, which could serve as a reservoir for various metabolic necessities. To test this, Myc-CaP and TRAMP-C2 animals were given L1 (150 mg/kg, daily gavage) starting 1-week post-orthotopic tumor implantation and continued until control tumors reached approximately 1–2 cm^3^ as measured by MRI (Fig. 4a). Prostate tumor volumes were significantly smaller in L1-treated Myc-CaP animals (p < 0.01) compared with control mice, and consistent with our hypothesis, L1 exhibited a higher efficacy in the low-iron TRAMP-C2 models determined by comparison of endpoint volumes (Fig. 4b, p < 0.001). Iron MRI assessments of tissue iron levels did not change in a systematic manner between models; only small increases in iron MRI distribution medians were observed in TRAMP-C2 spleens with L1 (P < 0.05, Fig. 4c), and nominal reductions in liver iron levels were observed in the Myc-CaP model (p < 0.05, Fig. 4d). Changes in fer granules were evaluated from Prussian blue histology and showed that while in TRAMP-C2 levels remained low and unchanged with L1 (p > 0.05, Fig. 4e), prostate tumor granules were significantly reduced (p < 0.001) and spleen granules significantly increased (p < 0.01) with chelation in the Myc-CaP mice (Fig. 4f). Similarly total HLM counts made from whole tissue analysis of TRAMP-C2 (Fig. 4e) and Myc-CaP (Fig. 4f) did not exhibit changes in all organs (p > 0.05), but Myc-CaP prostate tumor HLMs exhibited an increase with L1 (p < 0.001). Total CD68^+^ macrophage counts (Fig. 4g,h) also went largely unchanged with the chelation treatment in all tissues of the models (p > 0.05). These analyses reveal that short courses of L1 have minimal systematic negative perturbing effects on cellular iron deposits in these cancer trials—even in the low-iron TRAMP-C2 model—and suggest that differential levels of iron accumulation, rather than changes in sub-voxel iron distributions are associated with efficacy of chelation therapy.

**Figure 5:**
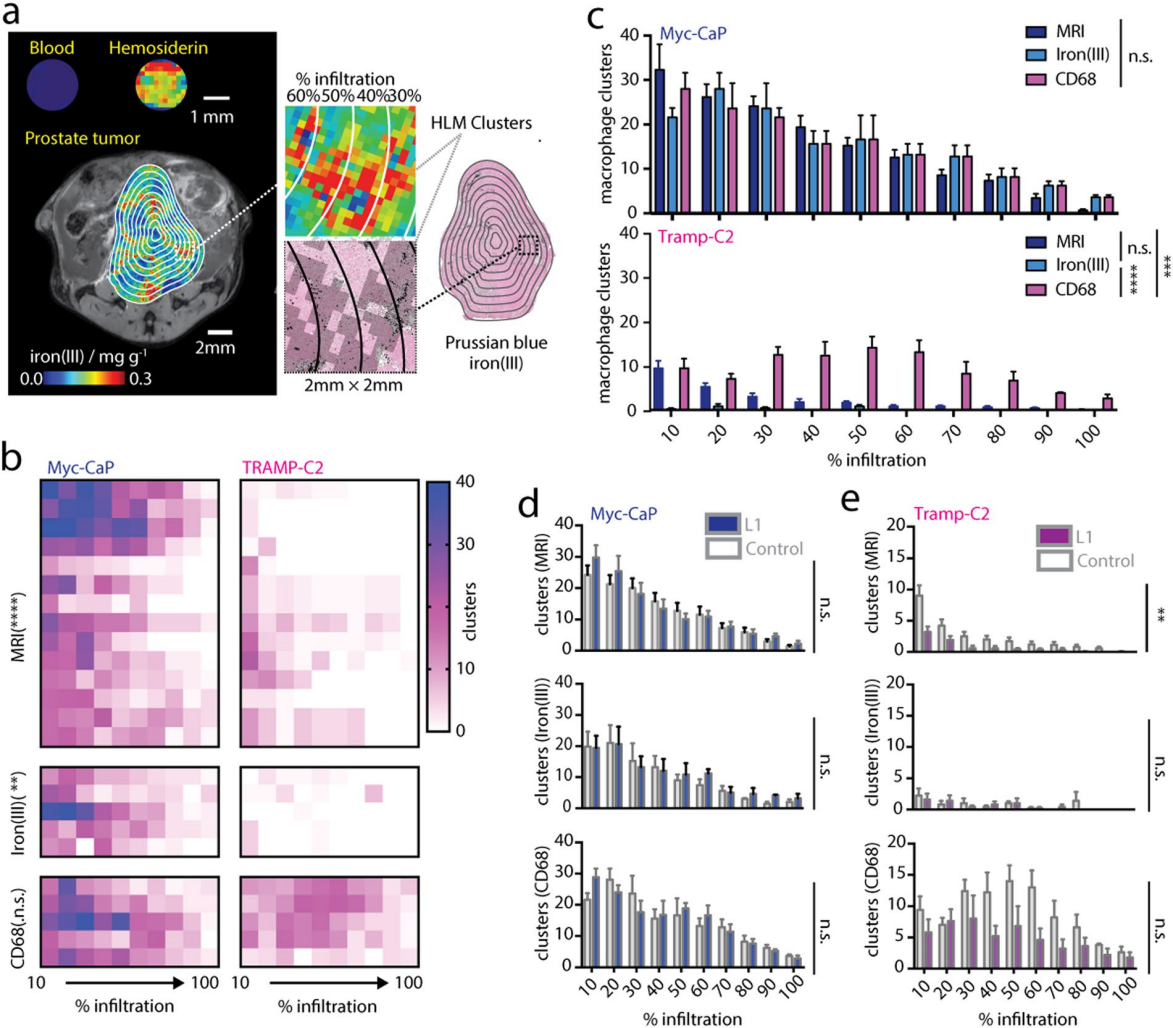
Mapping macrophage infiltration and iron deposition in chelation cancer therapy. (**a**, left) Iron MRI maps of blood, hemosiderin, and *in vivo* in prostate tumors analyzed beside (right) Prussian blue iron(III) stained whole tumor tissue cross-sections. Expansions show hemosiderin-laden macrophage (HLM) regions (MRI, colormap; histology, solid black masking) and MRI resolution-matched clusters in the Prussian blue histology (grayscale overlay) together with the concentric decile counting regions used to determine % macrophage infiltration of the clusters in tumors (outlines). Expansion box scale 2 mm × 2 mm. (**b**) Heatmaps of macrophage cluster infiltration. Asterisks refer to statistical comparisons of infiltration profiles between models (MRI n = 14 mice per model; histology n = 5 sections per model; n.s. P > 0.05, **p < 0.01, ****p < 0.0001 2-way ANOVA). (**c**) Statistical comparison between MRI, resolution-matched iron(III) histology, and resolution-matched CD68 infiltration profiles for (upper) and TRAMP-C2 (lower) tumors (MRI n = 14 mice per model; histology n = 5 sections per model; n.s. P > 0.05, **p < 0.01, ****p < 0.0001 2-way ANOVA). Infiltration profiles for control and L1-treated (**d**) Myc-CaP and (**e**) TRAMP-C2 tumors measured from (upper) high-iron(III) MRI maps, (center) resolution-matched Prussian blue iron(III) histology, and (bottom) resolution-matched CD68 histology (MRI n = 14 mice per model; histology n = 5 sections per model; n.s. P > 0.05, **p < 0.01 2-way ANOVA).

We then assessed whether the spatial distributions of HLMs are also associated with chelation response, and whether iron MRI can be used as a probe for localized macrophage infiltration *in vivo*. The sensitivity of the iron MRI method for different bio-iron types was tested by imaging blood, and HLMs isolated from spleen tissue and showed a clear differentiation between heme (low-iron) and non-heme hemosiderin iron (high-iron) sources (Fig. 5a). Using this observation, re-inspection of the iron(III) MRI maps indeed revealed localized clusters of high-iron(III) pixels (total range 0–0.3 mg g^−1^, high-iron(III) 0.15–0.3 mg g^−1^) which were confirmed to be deposits of iron(III)^+^ HLMs in the Prussian Blue histology. MRI maps were stratified in the high-iron range, and these high-iron(III) MRI clusters quantified algorithmically, and compared to a similar cluster detection and quantification procedure applied to iron(III)^+^ or CD68 histology using MRI resolution-matched histological images of the tumor cross-sections (Fig. 5a). We assessed the similarity between the measurements of HLM clusters by counting them in concentric decile regions of interest projected over the MRI or histological cross-sections, and graphing them as a function of their tumor infiltration (10% outer edge, 100% core). Infiltration profiles of the clusters generated from high-iron(III) MRI images and resolution-matched iron(III) and CD68 macrophage cluster maps in the prostate tumors (Fig. 5b) indicated significant differences between Myc-CaP and TRAMP-C2 animals as measured from MRI (p < 0.0001), iron histology (p < 0.01), but not CD68 histology alone (p > 0.05). Comparisons between the various methods (Fig. 5c) showed that in Myc-CaP tumors, high-iron(III) MRI, Prussian blue iron(IIII)^+^ HLM and CD68^+^ macrophage cluster maps exhibited similar quantities and degree of infiltration of the clusters (P > 0.05), while in the TRAMP-C2 model the HLM cluster infiltration profiles mapped by MRI were also the same as those reconstructed from iron(III)^+^ macrophage clusters (p > 0.05), but CD68^+^ macrophage infiltration profiles were significantly different from those mapped by MRI (p < 0.001) and Prussian blue histology (p < 0.0001). Macrophage infiltration profiling was then conducted in the L1 trial showing that the large differences in HLM infiltration between models persisted following L1 treatment. Macrophage cluster tumor infiltration in Myc-CaP models exhibited a nominal but not significant (p > 0.05) increase indicating that changes in iron content of macrophages occurred within clusters rather than forming new deposits, while in TRAMP-C2 models macrophage infiltration was reduced in the iron(III) and CD68 clusters but only MRI measurements were associated with statistically significant reductions in these clusters (p < 0.01) with L1 chelation therapy (Fig. 5d,e). These measurements validate a unique approach for the spatial mapping of macrophage infiltration using standard MRI approaches using the iron content of tumor macrophages as an endogenous contrast source, and further suggests that higher levels of HLM infiltration are a barrier to chelation efficacy for reducing tumor growth in prostate cancer.

## Discussion and Conclusions

The measurements presented point to a role of iron(III) in influencing both spatial and phenotypic distributions of infiltrating macrophages in prostate tumors. Compared to the general macrophage populations found in the tumor, the high-iron(III) macrophages exhibit unique model-specific infiltrative behavior. In the high-iron(III) Myc-CaP mouse model, the iron(III)^+^ macrophages and the CD68^+^ macrophages were found abundantly in reticuloendothelial organs (liver and spleen), and densely infiltrated the tumor cross-sections. By contrast, HLMs were scarce in similar TRAMP-C2 tissues, and only superficially infiltrated tumors (cf. Figure 2). Although the iron(III)^+^ macrophages were few in this model, the CD68^+^ macrophages not exhibiting the iron(III) phenotype were readily detected in the tumors and other organs and infiltrated the tumor extensively (cf. Figure 4). This indicates that while the two models are similarly immune competent, strain background impacts iron distribution systemically^50^, and in these measurements, such differences in iron metabolism are related to the abundance and infiltration of macrophages in the tumors.

Chelation of iron(III) has been proposed as a cancer treatment that causes reductions in tumor growth by decreasing the bioavailability of iron(III) to the proliferating cancer cells resulting in cytostatic effects^17–19, 26–29^. Both Myc-CaP and TRAMP-C2 exhibited significant anti-tumor response to L1 treatment that points to the drug having an effect after a short duration, over the course of weeks of tumor growth. Our imaging studies however provided few indications of cellular iron depletion with administration of L1 as cancer therapy, an observation consistent with the expected safe effects on cellular iron observed by MRI in clinical settings following months-years chelation regimens^52^. This indicates that while long-term chelation of cytotoxic free iron(III) can disrupt cellular deposition of non-heme iron leading to its eventual depletion over years, short term L1 administration is able to exert anti-tumor effects without significant perturbation to non-heme iron accumulation in these models. Thus, our measurements suggest that rather than changes in HLM infiltration being a therapeutic biomarker of cancer chelation response, the level of HLM accumulation measured both in tumors and in livers and spleens can be used as pre-treatment measures of expected chelation efficacy.

The current study represents the first imaging assessments of the distributions of non-heme cellular iron sources in orthotopic prostate tumors alone, together with systemic tissue measurements in cancer models. By confirming that the iron(III) deposition occurs predominantly in HLMs, and that the *in vivo* MRI contrast is assignable to clusters of these iron-laden cells by utilizing image analysis methods to map the HLMs as a function of HLM cluster infiltration, we could provide unbiased quantitative measures of macrophage deposition *in vivo*. Further, in the context of iron chelation therapy, imaging HLMs provided a correlation between the levels of iron-laden macrophage infiltration and anti-tumor therapeutic response, suggesting that this spatially resolved immune biomarker can also potentially be used in predictive manner for cancer chelation therapy. Taken together, imaging macrophage infiltration according to innate iron status provides a surrogate “biopsy” for monitoring macrophages that links iron deposition with systemic and tumoral accumulation of macrophages that is a prognostic biomarker associated with chelation therapy response.

## Methods

### MRI

All MRI images were acquired with a 7 T/30 cm horizontal bore Bruker Biospec MRI system with a custom-built 30 mm inner-diameter transmit-receive radio-frequency quadrature coil. Following standard adjustments, field homogeneity optimization was performed using the Bruker Mapshim algorithm. A 2D multi-gradient echo (MGE) relaxometry pulse sequence was used to acquire multi-echo T_2_^*^ relaxometry images with the following parameters: 16 evenly spaced TE’s (3.5 ms), TR 3 s, matrix 256 × 256 with 49 axial slices, in-plane spatial resolution 0.1 mm × 0.1 mm with slice thickness 0.5 mm, RF flip angle 90^°^, and each phase encode was gated on the animal’s respiration. Pixel-by-pixel determination of T_2_^*^ relaxation times was performed by fitting the magnitude of the MGE image series with a standard bias corrected mono-exponential function in Matlab (Natick, MA) and Fiji^53, 54^. No multi-exponential behavior was observed in the tissues studied. A series of aqueous iron(III) solutions were prepared by dissolving Fe^3+^(NO_3_^−^)_3_ (Fisher Scientific) within the concentration range of 0.0–0.3 mg iron(III) g^−1^ and used as the reference iron(III) range. A linear relation between the relaxation rate R_2_^*^ = 1/T_2_^*^ and iron(III) concentration was found, and was subsequently used to generate all parametric iron(III) MRI maps. Iron(III) MRI maps were stratified by high concentration (total range, 0.0–0.3 mg g^−1^_;_ high, 0.15–0.3 mg g^−1^) to generate high-iron pixel cluster maps. Spatial distributions of the high-iron(III) pixel clusters were characterized by performing cluster analysis over whole tissue cross-sections using the Fiji Analyze Cluster tool. Infiltration profiling of these high-iron(III) MRI clusters was conducted automatically by projecting concentric decile regions over the entire tumor cross-section, and counting the high-iron clusters within each of these regions in ImageJ.

### Animal Models

Animal experiments were approved by MSKCC IACUC committee and performed in accordance with their guidelines and regulations. Male 5–6 week C57BL/6 and FVB/N mice were procured from Charles River Laboratories and housed in the MSKCC vivarium and maintained under normal conditions. TRAMP-C2 cells^55, 56^ were grown in Dulbecco’s Modified Eagle’s (DME) medium, containing 25 mM glucose, 4 mM glutamine, 1% penicillin/streptomycin (Gibco), 5% fetal bovine serum and 5% Nu-serum IV (BD Scientific), 0.86 μM human insulin (Gibco), 10 mM 4-(2-hydroxyethyl)-1-piperazineethanesulfonic acid (HEPES) buffer, and 10 nM dihydrotestosterone (Steraloids Newport, RI, USA). Myc-CaP cells^57^ were grown in DME medium (5.6 mM glucose and 4 mM glutamine), supplemented with 10% fetal bovine serum and 1% penicillin/streptomycin (Gibco). Both cell lines were grown in 5% CO_2_/95% air (21% O_2_) at 37 °C in a humidified chamber, split every two days and used at passage 2–3. Six-week old mice underwent intraprostatic injection with 20 µL of 5 × 10^4^ PCa cells suspended in PBS and growth factor reduced phenol-red free ECM gel at a 70% vol./vol. concentration (Sigma-Aldrich). Animals received 150 mg deferiprone kg^−1^ (Sigma-Aldrich) by oral gavage in distilled water daily, 5 days per week beginning 1 week post tumor cell implantation and continuing until the control tumors reached approximately 1–2 cm^3^, approximately 4 weeks and 10 weeks in the Myc-CaP and TRAMP-C2 models, respectively. Mice were anesthetized with 1–3% isoflurane in O_2_ gas, and respiration was monitored during all imaging sessions. Blood was withdrawn by vein puncture and collected in heparinized tubes. Crude hemosiderin was obtained from fresh mouse spleen by magnetic separation using MACS columns and running buffer (Miltenyi).

### Histology

Sections of PBS-perfused tissue were collected and fixed in 4% PFA for 24 hours at 4 °C and then washed with H_2_O and re-suspended in 70% ethanol (Fisher Scientific). Tissues were paraffin embedded and 5 μm sections cut onto glass slides.

The Prussian blue histochemical iron(III) assays were performed at the Molecular Cytology Core Facility of MSKCC. Slides were manually de-paraffinized in xylene, re-hydrated in series of alcohol dilutions (100%, 95% and 70%) and tap water. Slides were placed in a working solution of equal parts 5% potassium ferricyanide (Fisher Scientific) and 5% hydrochloric acid (Fisher Scientific) prepared in distilled water and stained for 30 minutes. Slides were then rinsed in distilled water, counter-stained with nuclear-fast red and cover-slipped with Permount (Fisher Scientific).

The immunohistochemical detection of CD68, was performed at the Molecular Cytology Core Facility of MSKCC using a Discovery XT processor (Ventana Medical Systems). The tissue sections were deparaffinized with EZPrep buffer (Ventana Medical Systems), antigen retrieval was performed with CC1 buffer (Ventana Medical Systems) and sections were blocked for 30 minutes with Background Buster solution (Innovex) followed by avidin/biotin blocking for 8 minutes. CD68 (rabbit polyclonal antibody, Boster, cat# PA1518, 5 ug/ml) was applied and sections were incubated for 5 hours, followed by 60 minutes incubation with biotinylated goat anti-rabbit antibodies (Vector Labs, cat#PK6101) at 1:200 dilution. The detection was performed Streptavidin- HRP and DAB (DAB detection kit from Ventana Medical Systems) according to manufacturer’s instructions. Slides were counterstained with hematoxylin and cover-slipped with Permount (Fisher Scientific).

Histological sections were digitized with a Mirax Scan system and read with Panoramic Viewer (3DHISTECH, Budapest Hungary). Images were first visually inspected, and then the whole images were exported with the Panoramic Viewer at 1:4 pixel resolution in 256 × 256 tiles. These images were then imported and quantified by Fiji image processing macros. Images were thresholded to identify cells based on their labeling. Iron(III)^+^ particles (ferritin granules and HLMs) were identified by their blue color, and CD68^+^ macrophages were identified by their brown DAB color. The resulting image masks were used in the subsequent quantitative analysis. Modeling bi-exponential size distributions and linear correlation of number of iron particles with median MRI iron(III) pixel levels was performed using standard Matlab fit functions. We modeled the biexponential (small fer, large HLM) size distributions using the function y = a × exp(−bx) + c × exp(−dx), where y is the number of iron(III)^+^ particles, a and c are the numbers of small and large cellular iron(III) particles, b and d are the size constant of the fer and HLM particles, respectively, and x are the measured sizes of the particles. For CD68 y = a × exp(−bx), where y is the number of CD68^+^ macrophages, a the number of all CD68^+^ cells and b the size constant of their exponential distribution, and x the sizes of all CD68^+^ cells. Resolution-matched histology was generated by resizing the histological images (Prussian blue iron(III), CD68) by using pixel averaging and bilinear interpolation in ImageJ to down-sample the image size (1:100) to the resolution of the MRI experiment. The resulting resolution-matched images displayed “clusters” akin to the stratified high-iron MRI cluster masks, and were further discretized by watershed gradient processing, and spatial characteristics of the clusters were determined using the Fiji Analyze Cluster tool. Infiltration profiling of the resolution-matched clusters was conducted by projecting concentric decile regions over the entire tumor cross-section, and counting the resolution-matched clusters them within each of these regions with ImageJ.

### Statistics

Data was analyzed using two-tailed unpaired t-tests for normal distributions; Mann-Whitney tests for non-Gaussian distributions, or 2-way analysis of variance (ANOVA) for multivariate statistical analysis all in GraphPad Prism 7.
